# Supplementary figures and images for: In silico analysis of quorum sensing modulators: Insights into molecular docking and dynamics and potential therapeutic applications
Source: PLoS One. 2025 Jun 9;20(6):e0325830. doi: 10.1371/journal.pone.0325830 (PMC12148138; doi:10.1371/journal.pone.0325830)

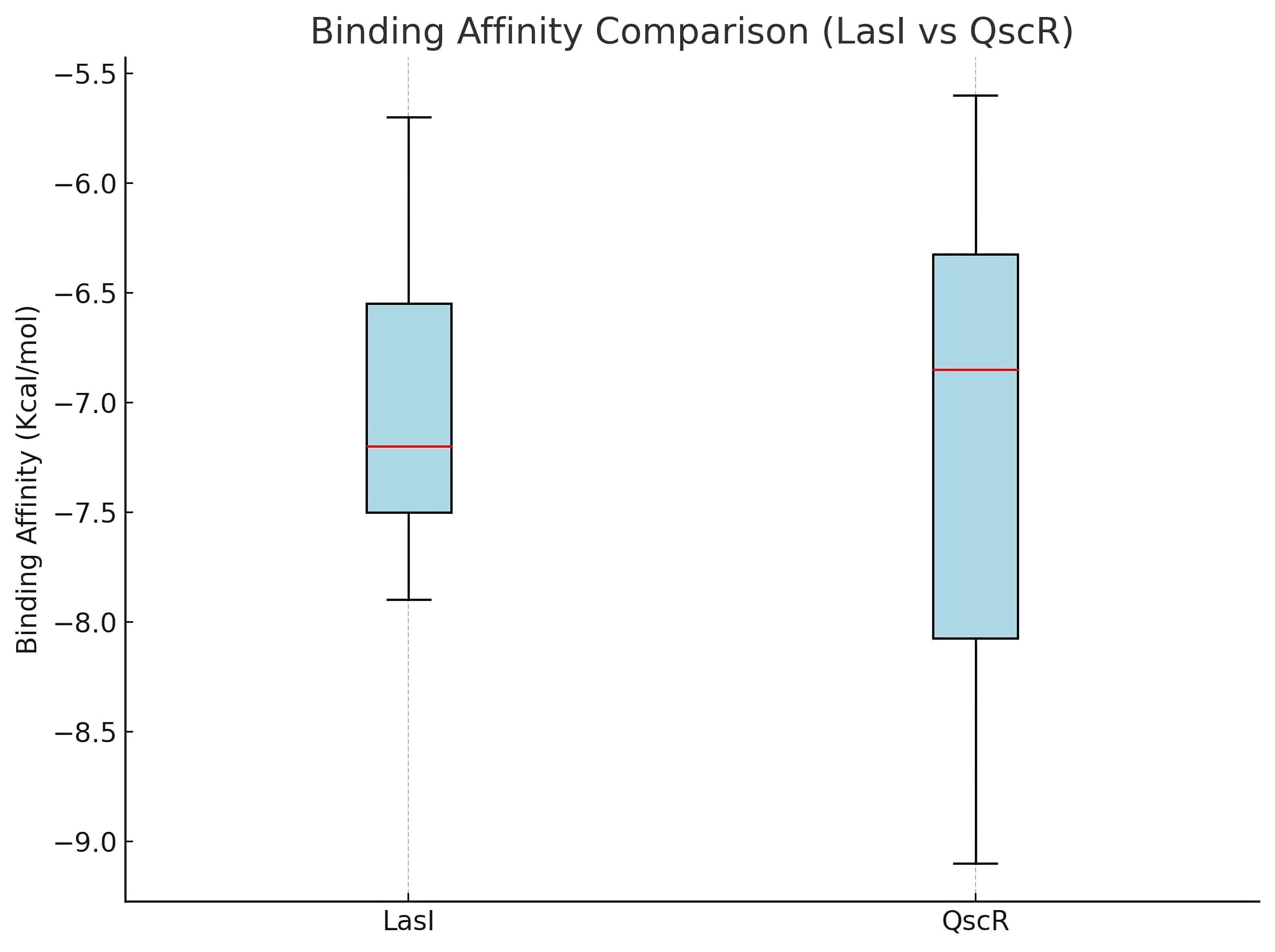

Supplement: S1 Fig — The median binding affinities are represented by red lines within the boxes. While QscR shows a broader distribution of affinities, statistical analysis using the Mann–Whitney U test (U = 104.0, p = 0.800) indicates no significant difference between the two groups (p > 0.05), suggesting comparable binding tendencies of the compounds toward both targets. (PNG) [file pone.0325830.s001.png]

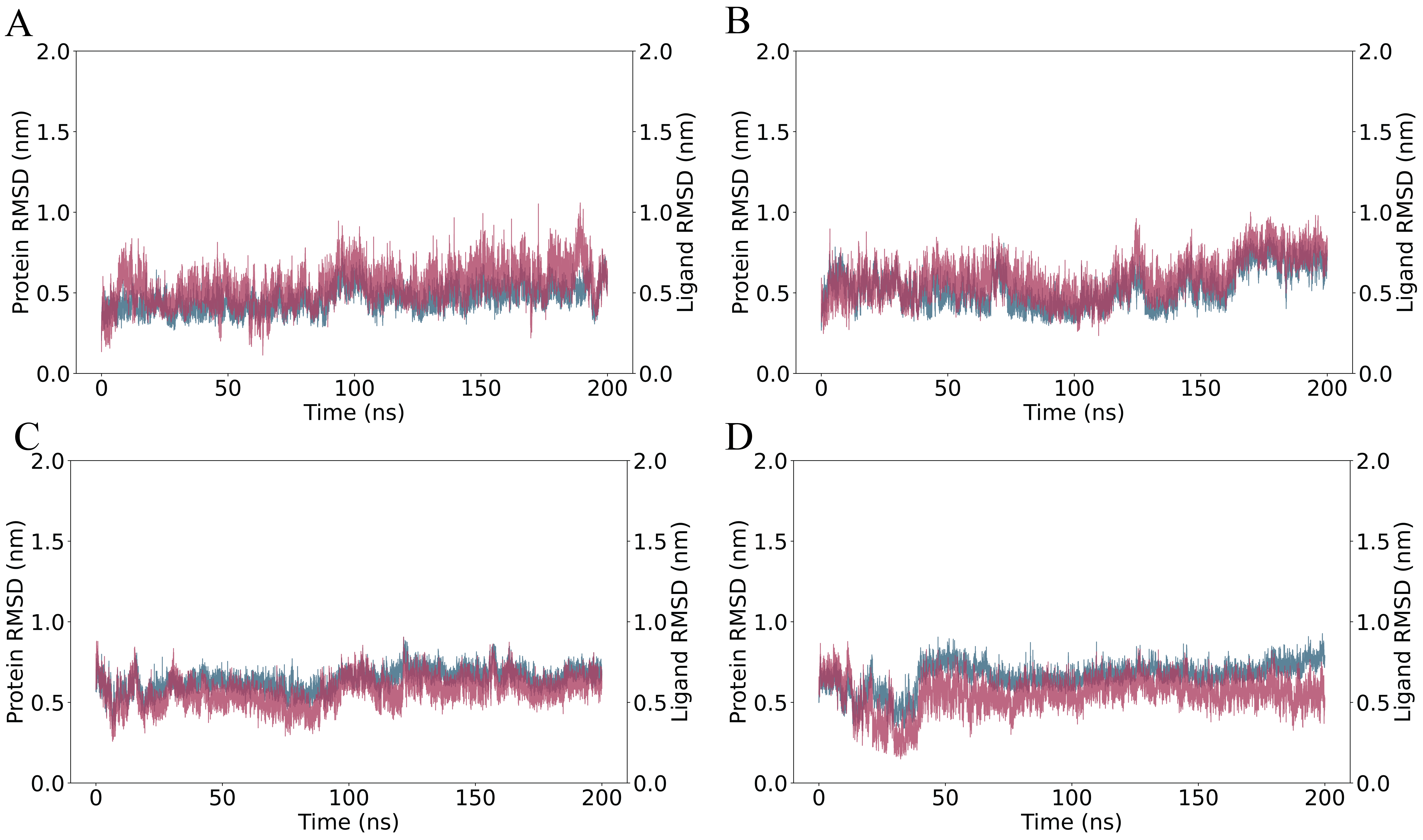

Supplement: S2 Fig — Root Mean Square Deviation (RMSD) analysis for LasI complexed with Quercetin (CID 5325), LasI with Ginkgolide A (CID 68933), QscR with Chloro-N-(4-fluorobenzyl)thiophene-2-sulfonamide (CID 893742), and QscR with N-(carbamoylcarbamothioyl)-2-chlorobenzamide (CID 2796468). Protein RMSD (cyan) and ligand RMSD (red) are plotted, with the X-axis representing time (nanoseconds) and Y-axis for RMSD (nanometers). Results demonstrate stable complexes with minimal fluctuations. (PNG) [file pone.0325830.s002.png]

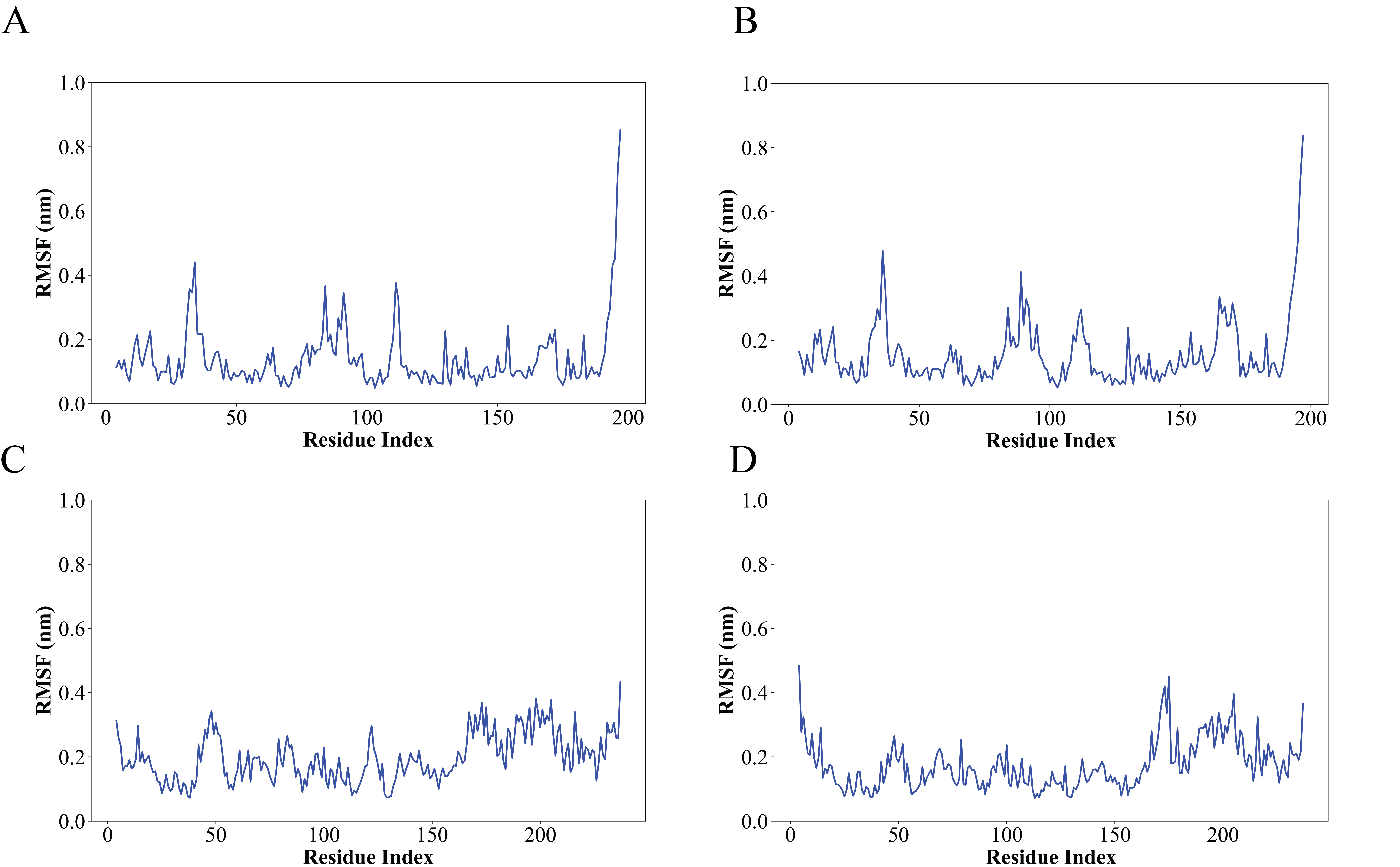

Supplement: S3 Fig — RMSF analysis of protein-ligand complexes over a 200 ns simulation. Root Mean Square Fluctuation (RMSF) analysis highlighting the flexibility of residues in each protein-ligand complex. Specific regions with notable fluctuations are identified, such as residues 100–150 in the LasI-Quercetin complex and residues 25–50 in other complexes, reflecting ligand influence on protein dynamics. (PNG) [file pone.0325830.s003.png]

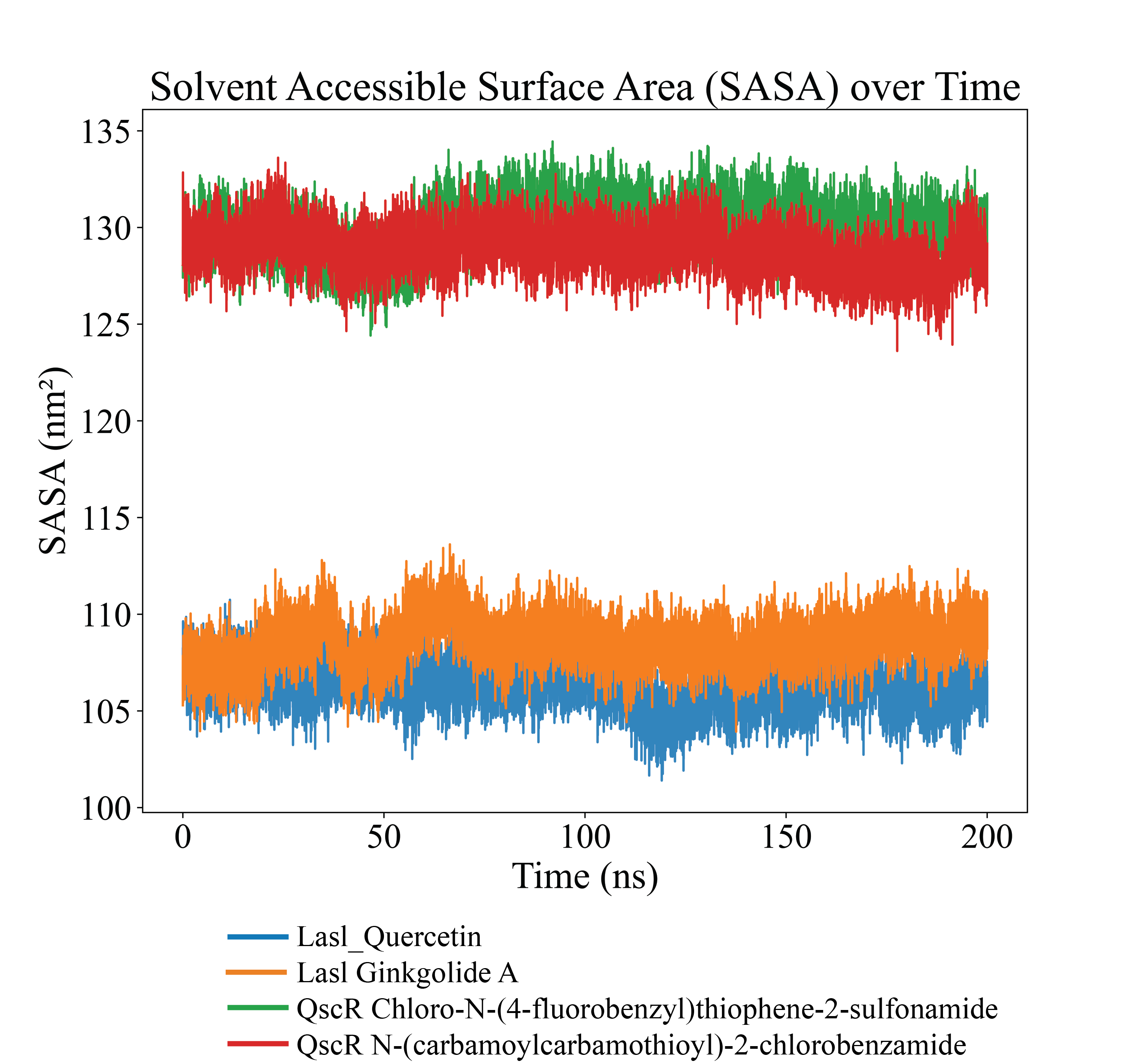

Supplement: S4 Fig — Solvent Accessible Surface Area (SASA) analysis showing differences in solvent exposure among the complexes. LasI-Quercetin exhibited the highest SASA values (130–135 nm²), while QscR-Chloro-N-(4-fluorobenzyl)thiophene-2-sulfonamide displayed lower SASA values (100–110 nm²). (PNG) [file pone.0325830.s004.png]

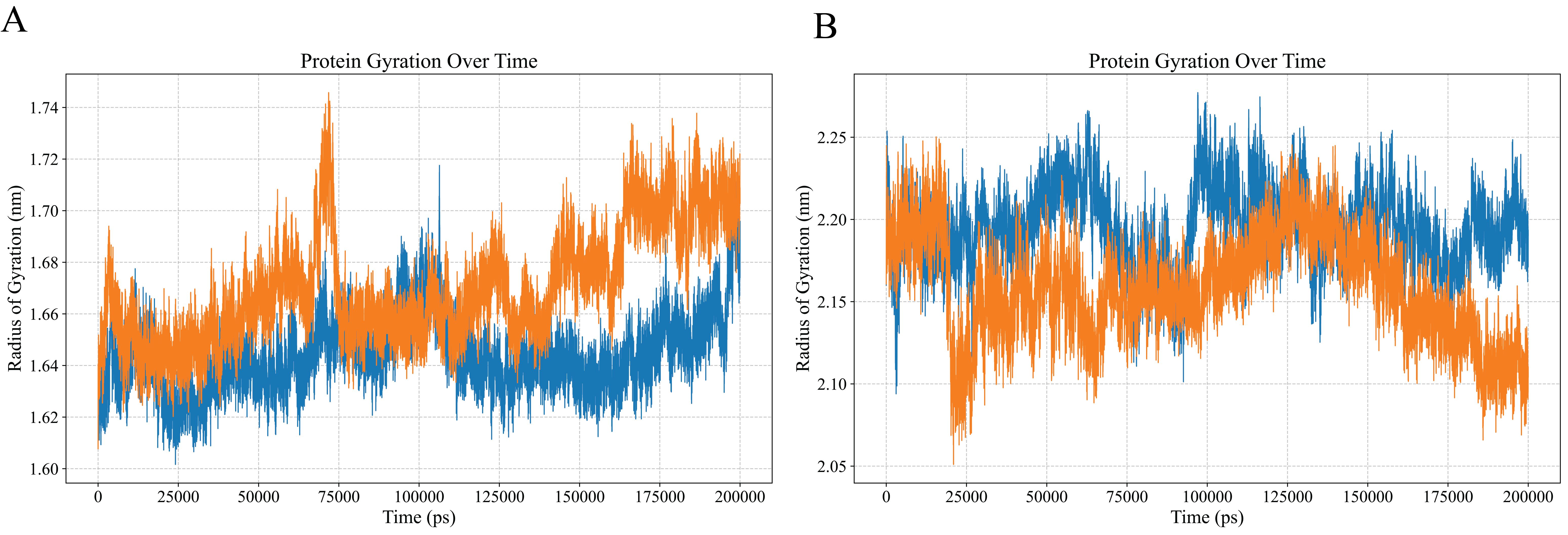

Supplement: S5 Fig — Radius of Gyration (Rg) analysis illustrates the compactness of the complexes. LasI-Quercetin complex remained stable (1.65–1.7 nm), while QscR-N-(carbamoylcarbamothioyl)-2-chlorobenzamide showed wider Rg variations (2.05–2.25 nm), indicating conformational flexibility. (PNG) [file pone.0325830.s005.png]
