# Supplementary material for: In silico analysis of quorum sensing modulators: Insights into molecular docking and dynamics and potential therapeutic applications
Source: PLoS One. 2025 Jun 9;20(6):e0325830. doi: 10.1371/journal.pone.0325830 (PMC12148138; doi:10.1371/journal.pone.0325830)
Supplement: Attachment — Submitted filename: 4.pdf [file pone.0325830.s008.pdf]

The manuscript presents a comprehensive in silico analysis of quorum sensing (QS) modulators, focusing on the structural dynamics of LasI and QscR proteins in *Pseudomonas aeruginosa*. The study employs molecular docking and molecular dynamics (MD) simulations to evaluate the binding affinity and stability of potential QS inhibitors (QSIs), with a particular focus on Sulfamerazine and Sulfaperin. The manuscript is well-structured and addresses an important area of research, given the rising concern of antimicrobial resistance (AMR) and the need for alternative therapeutic strategies. However, there are several areas where the manuscript could be improved to enhance its scientific rigor, clarity, and impact.

- While the in-silico approach is valuable for initial screening, the absence of experimental validation (e.g., in vitro assays) limits the study's impact. The authors should acknowledge this limitation more explicitly and discuss plans for future experimental validation, such as bacterial quorum sensing assays (e.g., Chromobacterium violaceum CV026 bioassay) or virulence factor inhibition assays in *P. aeruginosa*.
- The manuscript does not provide a strong rationale for selecting Sulfamerazine and Sulfaperin as the primary ligands. While the ADMET profiling is thorough, more context is needed regarding why these specific compounds were chosen over others. For example, are these compounds known to have antimicrobial or QS inhibitory properties in previous studies?
- The discussion section could be expanded to include a more critical evaluation of the study's findings in the context of existing literature. For instance, how do the binding affinities and stability of Sulfamerazine and Sulfaperin compare to other known QSIs? Additionally, the authors should discuss the potential challenges in translating these findings into clinical applications, such as bioavailability, toxicity, and resistance development.
- The use of AliA lactonase as a negative control is somewhat confusing. While the authors explain that AliA was used for reference docking, it is not clear how this serves as a negative control in the context of MD simulations. A more appropriate negative control would have been a known non-inhibitor or a scrambled peptide. This should be clarified in the manuscript.
- The manuscript mentions that docking simulations were performed in triplicate, but there is no detailed statistical analysis to support the robustness of the results. Including statistical tests (e.g., Mann-Whitney U test, Shapiro-Wilk test) would strengthen the validity of the findings.
- The study focuses exclusively on AHL-based QS systems in Gram-negative bacteria. While this is justified, the authors should briefly discuss the potential applicability of their findings to other QS systems (e.g., AI-2 or AIP systems) and Gram-positive bacteria. This would broaden the scope and relevance of the study.
