# Supplementary material for: In silico analysis of quorum sensing modulators: Insights into molecular docking and dynamics and potential therapeutic applications
Source: PLoS One. 2025 Jun 9;20(6):e0325830. doi: 10.1371/journal.pone.0325830 (PMC12148138; doi:10.1371/journal.pone.0325830)
Supplement: Attachment — Submitted filename: PLOS One Alisaac - review comments.docx [file pone.0325830.s006.docx]

Title: In Silico Analysis of Quorum Sensing Modulators: Insights into Molecular Docking and Dynamics and Potential Therapeutic Applications

Should: investigate structural dynamics of quorum sensing proteins LasI and QscR in complex with various ligands to identify potential molecules and scaffolds for use as AHL quorum sensing inhibitors

Did: show in silico studies that indicate LasI and QscR bind with sulfaperin and sulfamerazine

Manuscript Comments

Introduction:

- Appropriate justification for investigating quorum sensing inhibitors (QSI)
- 2^nd^ paragraph: This could be said more succinctly by discussing ESKAPE pathogens in general. I'm not sure why *Pseudomonas* is being singled out as different from the other ESKAPE pathogens here.
- 3^rd^ and 4^th^ paragraphs: The author has made no indication that they recognize that these bacterial species do not all have the same QS system machinery, particularly grouping the gram positive *S. aureus* in with the gram negative species mentioned. Additionally, they should point out LasI or LuxI-type homolog systems in *E. coli* or *K. pneumoniae* to justify the broad-spectrum potential of this QSI search.
- 5^th^ paragraph: More justification for the focus on LasI and QscR should be provided. For instance, what virulence factors do they regulate? Are the systems universal or are there homologous systems in non-pseudmonads? Also, an agr-like system would be needed to extend these in silico results to *S. aureus*.
- “By investigating the potential of QSIs, this research seeks to provide a foundation for novel treatments that could complement existing antibiotics and improve patient outcomes in the battle against antibiotic-resistant infections.”: **There already exists a broad foundation for quorum sensing inhibitors; this could certainly add to it, but the author hasn't acknowledged previously identified QSI. Why is this study particularly different from previous efforts to identify QSI?**

Methods:

- Selection of ligand
  - Why were phytochemicals particularly explored? It seems unclear if there was a reason to focus on phytochemicals, especially when the author indicates other classes of chemicals were screened. Additionally, if the final compounds investigated are phytochemicals, this should be indicated in the discussion.
  - The author should further describe “pertinent information” or indicate where that information can be found elsewhere in the text.
    - This goes for “the chosen compounds were selected for further analysis” as well.
- Protein Selection:
  - Which structures came from *P. aeruginosa* and which came from *B. thuringiensis*?
  - I am confused on how AiiA lactonase is a negative control. If it disrupts QS, would you not have to show how it interacts with the QS proteins? How does a QS disruptor complexing with your ligands act as a control for how your ligands bind to QS proteins? An appropriate positive control (ie. a known LasI or QscR inhibitor) should have been included for comparison.
- ADMET profiling, Molecular docking, and Molecular dynamic simulation sections: clearly explained and easy to follow methods

Results

- Are all ligands screened in a supplemental file? Author does not indicate.
- “The ADMET filtered 23 phytochemicals, and positive control was further analyzed through docking in PyRx (Table 3).”: **What was the positive control used for comparison in the docking assay? Because AiiA lactonase was defined as a negative control earlier in the text. Or does the author mean binding activity was analyzed (looking for a positive for interaction between protein and ligand)?**
- “Sulfaperin (CID 68933) and Sulfamerazine (CID 5325) have common proteins in both proteins could be possible as good binding stability”: **Unclear meaning - perhaps the author meant common amino acid motifs in the protein that suggest good binding stability? If that's the case, that motif should be discussed.**
- “Only the complexes that exhibited favorable RMSD, RMSF, SASA, and gyration results during the MD simulations were included for further analysis of the binding grooves.” : **Move this up one sentence (after "The simulation results were analyzed using various metrics, including RMSD, RMSF, SASA, and radius of gyration") , then include which ligands passed this threshold.**
- “…with the ligand stabilizing after 150 ns, suggesting a similar dynamic binding behavior to Lasl_68933 (Figure 4).”: **This is the first time the author has referred to a protein-ligand complex with just the ligand CID rather than the compound name - choose one way and keep it consistent. Also, Figure 4 should have a panel letter (4B).**
- “The other simulation that conduct based on the binding affinity Lasl with Quercetin (CID 5280343) and Ginkgolide A (CID 9909368) have also a better binding stability over the time and QscR with Chloro-N-(4-fluorobenzyl)thiophene-2-sulfonamide (CID 893742), and N-(carbamoylcarbamothioyl)-2-chlorobenzamide (CID 2796468) have also been shown better binding stability (Fig S1)” : **Rephrase as** “**Simulations based on the binding affinity of LasI with Quercetin (CID 5280343) and Ginkgolide A (CID 9909368) also have improved binding stability over the 200 ns analysis time. QscR with Chloro-N-(4-fluorobenzyl)thiophene-2-sulfonamide (CID 893742), and N-(carbamoylcarbamothioyl)-2-chlorobenzamide (CID 2796468) also show good binding stability in the MD simulations (Fig S1).**”
- “These observations indicate that while the protein exhibits inherent flexible regions.”: **This is an incomplete sentence – perhaps the author forgot to mention something about rigidity.**
- “In contrast, the QscR protein, when complexed with either Sulfamerazine or Sulfaperin, exhibits significantly higher Rg values, ranging from 2.10 nm to 2.30 nm. These values are notably higher than those observed for LasI, indicating a less compact structure in the QscR protein-ligand complexes.”: **Rephrase to “In contrast, the QscR protein, when complexed with either Sulfamerazine or Sulfaperin, exhibits significantly higher Rg values than LasI, ranging from 2.10 nm to 2.30 nm, indicating a less compact structure in the QscR protein-ligand complexes.”**
- “These differences in dynamic behavior may have implications for the functional properties of these complexes (Figure 9).”: **Implications should be discussed or this sentence should be removed.**

Discussion

- Most of this discussion reads like a brief summary of the results and methods. There is very little discussion of how this study fits into the larger search for quorum sensing inhibitors. There is no discussion about inhibition of QscR, and the author focuses on studies about LasR without relating them to the findings of their own study. A previously identified LasI inhibitor is mentioned, but it is not clear how that study relates to the in silico study conducted in the manuscript, as there is no mention of correlation of molecular similarities or dynamics. There is also very little discussion about activity of molecules similar to the ones investigated here (Sulfonamides).
  - Have sulfaperin and sulfamerazine been identified as QSI before? Have molecular dynamic studies identified other sulfonamides as QSI? Author simply goes into recent advances in QSI, nothing that would directly align with the present study.
- “In this study, we used molecular dynamics (MD) simulations to explore the structural dynamics of LasI and QscR proteins in complex 23 ligand, as well as a negative control, AiiA lactonase.”: **There was no mention of this as a control in the sections where the MD results were discussed. AiiA was only mentioned in the binding affinity studies as a metric for determining binding specificity. No interactions between AiiA and either QS protein or ligands were modeled in the simulations.**
- “Future experimental validation, using techniques like crystallography or surface plasmon resonance, could confirm the computational findings and help develop more potent QS inhibitors for therapeutic applications.”: **Future experimental validation should also include conducting studies in bacteria, such as with the *Chromobacterium violaceum* assay or measurement of QS regulated gene expression (ie. rhamnolipid production, pyocyanin production, etc.), to prove in vitro efficacy.**

Grammar/Typos:

- Check that “LasI” has an upper case “i” and not a “1” or lower case “L”. It appears incorrectly as either “Las1” or “Lasl” throughout the manuscript.
- Check bacterial names for italicization.
- Missing periods at the end of a couple paragraphs throughout the manuscript.
- Numbers are missing a few places (Results section 1 – corresponding to molecular weight near g/mol)
- Reference Figures before going into in depth discussion about what they show.
- There is an extra figure 7 caption right after the supplemental figure captions.

Summary: QS is a promising target to reduce antibiotic resistance in bacterial pathogens. The author conducted a screen of publically available molecular data to identify compounds with strict ADMET requirements that would potentially pass future in vivo efficacy and toxicity tests. Molecules that met those requirements were assessed for binding affinity and molecular dynamics in complex with two prominent QS proteins in *P. aeruginosa,* LasI and QscR. Sufalperin and sulfamerazine were the two best candidates, and showed strong binding affinity for LasI and QscR. The author suggests these would be good scaffolds for future drug discovery.

Overall impression: The screen, molecular docking, and molecular dynamics data indicate that the lead compounds deserve further investigation as QSI scaffolds. However, the lack of proper controls, lack of explanation of target selection, and lack of in vitro correlations leaves me wondering how effective these compounds will be down the line.

Major concerns:

- Negative control AiiA lactonase only acts as a metric for non-specific binding in in silico docking studies and there is no positive control of a previously identified LasI or QscR QSI. Therefore we have no way of ranking or quantifying just how effective these compounds might be as QSI in in vitro or, ultimately, in clinical use.

Minor Concerns:

- Needs more justification of why LasI and QscR particularly were investigated. I would also appreciate some reasoning as to why AHL systems were the only QS system targeted, rather than including AI-2 and AIP systems, especially when the introduction mentions the study aims to “identify potential quorum sensing inhibitors (QSIs) that can effectively disrupt the QS pathways of various bacterial species, including *P. aeruginosa*, *E. coli, Klebsiella pneumoniae*, and *Staphylococcus aureus*.” This could be modified by removing the mention of *S. aureus* and indicating a focus on AHL QS given its prevalence among gram negative species. Acknowledgement of the other systems would also be appreciated.
