# Supplementary material for: In silico analysis of quorum sensing modulators: Insights into molecular docking and dynamics and potential therapeutic applications
Source: PLoS One. 2025 Jun 9;20(6):e0325830. doi: 10.1371/journal.pone.0325830 (PMC12148138; doi:10.1371/journal.pone.0325830)
Supplement: Attachment — Submitted filename: Response to Reviewers file type.docx [file pone.0325830.s009.docx]

**Response to Reviewers**

**Manuscript Title:** *In Silico Analysis of Quorum Sensing Modulators: Insights into Molecular Docking and Dynamics and Potential Therapeutic Applications*

**Dear Editor and Reviewers,**

*We sincerely appreciate the time and effort you have taken to review our manuscript. We are grateful for the insightful comments and constructive feedback, which have helped us significantly improve the quality of our work. Below, we provide a detailed point-by-point response to each of the comments raised by the reviewers and the editor. Changes made in the manuscript are highlighted accordingly.*

**Response to Reviewer Comments**

**Introduction**

***Comment 1:****"Appropriate justification for investigating quorum sensing inhibitors (QSI)"*

**Response:** We appreciate the reviewer’s suggestion. We have strengthened the justification for investigating quorum sensing inhibitors (QSIs) by highlighting their potential in mitigating antimicrobial resistance without exerting direct selective pressure. We have revised the sentence:

**Revised Text: "Targeting bacterial quorum sensing (QS) has emerged as a promising strategy to mitigate antimicrobial resistance by disrupting coordinated behaviors such as virulence factor production, biofilm formation, and antibiotic resistance. Unlike traditional antibiotics, QS inhibitors (QSIs) interfere with bacterial communication without exerting direct selective pressure, reducing the likelihood of resistance development."**

***Comment 2:*** *"2nd paragraph: This could be said more succinctly by discussing ESKAPE pathogens in general. I'm not sure why Pseudomonas is being singled out as different from the other ESKAPE pathogens here."*

**Response:** Thank you for this valuable suggestion. We have revised this section to frame Pseudomonas aeruginosa within the broader context of ESKAPE pathogens, acknowledging quorum sensing as a key factor in the pathogenicity of all members of this group.

**Revised Text: "ESKAPE pathogens (Enterococcus faecium, Staphylococcus aureus, Klebsiella pneumoniae, Acinetobacter baumannii, Pseudomonas aeruginosa, and Enterobacter spp.) represent the leading causes of MDR infections worldwide, with quorum sensing playing a central role in their pathogenicity. While P. aeruginosa serves as a model organism for QS studies, similar regulatory systems contribute to virulence and antibiotic resistance in other ESKAPE pathogens."**

***Comment 3:****"3rd and 4th paragraphs: The author has made no indication that they recognize that these bacterial species do not all have the same QS system machinery, particularly grouping the gram-positive S. aureus in with the gram-negative species mentioned. Additionally, they should point out LasI or LuxI-type homolog systems in E. coli or K. pneumoniae to justify the broad-spectrum potential of this QSI search."*

**Response:** We appreciate respected Reviewer’s suggestion. We have explicitly acknowledged the differences between Gram-negative and Gram-positive quorum sensing systems and added references to LasI/LuxI homologs in E. coli and K. pneumoniae.

**Revised Text: "In Gram-negative bacteria such as P. aeruginosa, E. coli, and K. pneumoniae, QS is predominantly regulated by acyl-homoserine lactones (AHLs) through LasI/LuxI-type homologs. In contrast, Gram-positive bacteria like Staphylococcus aureus utilize an agr-based QS system that relies on autoinducing peptides (AIPs) instead of AHLs. Recognizing these mechanistic differences is crucial for designing broad-spectrum QS inhibitors."**

***Comment 4:*** *"5th paragraph: More justification for the focus on LasI and QscR should be provided. For instance, what virulence factors do they regulate? Are the systems universal, or are there homologous systems in non-Pseudomonads? Also, an agr-like system would be needed to extend these in silico results to S. aureus."*

**Response:** We have clarified the virulence factors regulated by LasI and QscR and discussed their homologous systems in non-Pseudomonas species. We also addressed the need for targeting an agr-like system in S. aureus.

**Revised Text: "LasI and QscR regulate key virulence factors in P. aeruginosa, including elastase, pyocyanin production, and biofilm formation. These proteins also share functional similarities with LuxI/LuxR-type systems in E. coli and K. pneumoniae, suggesting potential for broader application of QSIs. However, the extension of these findings to S. aureus would require targeting its agr system, which operates through a distinct autoinducer signaling mechanism."**

***Comment 5:****"By investigating the potential of QSIs, this research seeks to provide a foundation for novel treatments that could complement existing antibiotics and improve patient outcomes in the battle against antibiotic-resistant infections. There already exists a broad foundation for quorum sensing inhibitors; this could certainly add to it, but the author hasn't acknowledged previously identified QSI. Why is this study particularly different from previous efforts to identify QSI?"*

**Response:** We have acknowledged prior research on QSIs and emphasized how our study contributes new insights through molecular docking and MD simulations.

**Revised Text: "Quorum sensing inhibition has been explored as an alternative to conventional antibiotics, with numerous QSIs identified from natural and synthetic sources. This study builds upon previous efforts by integrating molecular docking and molecular dynamics simulations to assess ligand stability and binding specificity, providing mechanistic insights into QSI interactions with LasI and QscR. These computational analyses pave the way for experimental validation and potential therapeutic development."**

***Methods:***

**Selection of ligand**

***Comment 6:*** *Why were phytochemicals particularly explored? It seems unclear if there was a reason to focus on phytochemicals, especially when the author indicates other classes of chemicals were screened. Additionally, if the final compounds investigated are phytochemicals, this should be indicated in the discussion*

**Response:** Thank you for your valuable comment. We have clarified the reason for prioritizing phytochemicals in ligand selection. And additionally, we have mentioned in the discussion

**Revised Text: "Phytochemicals were chosen due to their established antimicrobial and quorum sensing inhibitory properties, as demonstrated in previous studies. Additionally, their structural diversity and biocompatibility make them attractive candidates for drug development. While other chemical classes were screened, phytochemicals were prioritized due to their potential for natural QS inhibition."**

***Comment 7:*** *"The author should further describe ‘pertinent information’ or indicate where that information can be found elsewhere in the text."* *This goes for “the chosen compounds were selected for further analysis” as well.*

**Response:** I clarified what "pertinent information" entails and explicitly referenced Table 3.

**Revised Text: "A thorough literature review was conducted to identify medicinal plants and their phytochemical constituents with documented antimicrobial or quorum sensing inhibition properties. This information included chemical structures, bioactivity profiles, and reported mechanisms of action. The botanical names of these plants were used as search terms in Dr. Duke's Phytochemical and Ethnobotanical Databases, and additional details regarding compound selection are provided in Table 3."**

***Protein Selection:***

***Comment 8:*** *"Which structures came from P. aeruginosa and which came from B. thuringiensis?"*

**Response:** We clarified the sources of the quorum sensing proteins by specifying their origins and providing relevant PDB IDs.

**Revised Text: "The crystal structures of quorum sensing proteins QscR (PDB ID: 6CC0) and LasI (PDB ID: 1RO5), both derived from Pseudomonas aeruginosa, and AiiA lactonase (PDB ID: 7L5F), derived from Bacillus thuringiensis, were retrieved from the RCSB Protein Data Bank."**

***Comment 9:*** *"I am confused on how AiiA lactonase is a negative control. If it disrupts QS, would you not have to show how it interacts with the QS proteins? How does a QS disruptor complexing with your ligands act as a control for how your ligands bind to QS proteins? An appropriate positive control (i.e., a known LasI or QscR inhibitor) should have been included for comparison."*

**Response:** We sincerely appreciate this insightful comment. We respectfully clarified that AiiA was included for reference docking rather than serving as a direct negative control in MD simulations.

**Revised Text: "While AiiA lactonase was initially included in docking analyses as a known quorum-quenching enzyme, it was not included in MD simulations. Therefore, it cannot be considered a direct negative control in this study. Instead, its docking results were used as a reference to compare the binding affinity of potential QSIs. Future studies should incorporate experimental validation to assess the functional inhibition of LasI and QscR by these ligands in bacterial quorum sensing models."**

***Comment 10: ADMET profiling, Molecular docking, and Molecular dynamic simulation sections: clearly explained and easy to follow methods***

***Response:*** Response: Thank you for your positive feedback. We appreciate that the ADMET profiling, molecular docking, and molecular dynamics simulation methods were found to be clearly explained and easy to follow. We have ensured that our methodology remains transparent and well-structured to facilitate reproducibility and interpretation. If any additional clarifications or details would further enhance these sections, we would be happy to incorporate them.

***Results Section***

*Comment 11: Are all ligands screened in a supplemental file? Author does not indicate.*

Response: Thank you for the suggestion. We have now clarified in the manuscript that all screened ligands and their ADMET properties are provided in the supplemental file (Supplementary CSV 1).

*Comment 12: “The ADMET filtered 23 phytochemicals, and positive control was further analyzed through docking in PyRx (Table 3).”: What was the positive control used for comparison in the docking assay? Because AiiA lactonase was defined as a negative control earlier in the text. Or does the author mean binding activity was analyzed (looking for a positive for interaction between protein and ligand)?*

Response: We appreciate the request for clarification. The term "positive control" was misleading, as no established inhibitors of LasI or QscR were available. We have revised the manuscript to clarify that AiiA lactonase was included as a reference quorum quenching enzyme, but no specific positive control was used.

*Comment 13: “Sulfaperin (CID 68933) and Sulfamerazine (CID 5325) have common proteins in both proteins could be possible as good binding stability”: Unclear meaning - perhaps the author meant common amino acid motifs in the protein that suggest good binding stability? If that's the case, that motif should be discussed.*

Response: We acknowledge the ambiguity and have rephrased the sentence to indicate that Sulfaperin and Sulfamerazine interact with conserved binding motifs in both LasI and QscR, which suggests favorable binding stability across these QS proteins.

*Comment 14: “Only the complexes that exhibited favorable RMSD, RMSF, SASA, and gyration results during the MD simulations were included for further analysis of the binding grooves.” : Move this up one sentence (after "The simulation results were analyzed using various metrics, including RMSD, RMSF, SASA, and radius of gyration") , then include which ligands passed this threshold.*

Response: We have moved the sentence regarding complex selection earlier in the paragraph, as suggested, and specified which ligand-protein complexes passed the selection criteria.

*Comment 15: “…with the ligand stabilizing after 150 ns, suggesting a similar dynamic binding behavior to Lasl_68933 (Figure 4).”: This is the first time the author has referred to a protein-ligand complex with just the ligand CID rather than the compound name - choose one way and keep it consistent. Also, Figure 4 should have a panel letter (4B).*

**Response:** Response: Consistency in referring to ligand-protein complexes has been ensured throughout the manuscript, and Figure 4 has been updated to include the appropriate panel designation (4B).

*Comment 16: “The other simulation that conduct based on the binding affinity Lasl with Quercetin (CID 5280343) and Ginkgolide A (CID 9909368) have also a better binding stability over the time and QscR with Chloro-N-(4-fluorobenzyl)thiophene-2-sulfonamide (CID 893742), and N-(carbamoylcarbamothioyl)-2-chlorobenzamide (CID 2796468) have also been shown better binding stability (Fig S1)” : Rephrase as “Simulations based on the binding affinity of LasI with Quercetin (CID 5280343) and Ginkgolide A (CID 9909368) also have improved binding stability over the 200 ns analysis time. QscR with Chloro-N-(4-fluorobenzyl)thiophene-2-sulfonamide (CID 893742), and N-(carbamoylcarbamothioyl)-2-chlorobenzamide (CID 2796468) also show good binding stability in the MD simulations (Fig S1).”*

Response: Thank You for your insightful comments. The suggested rephrasing has been incorporated to improve clarity in describing the simulation results

*Comment 17: “These observations indicate that while the protein exhibits inherent flexible regions.”: This is an incomplete sentence – perhaps the author forgot to mention something about rigidity.*

**Response:** The incomplete sentence has been revised to explicitly state that ligand binding contributes to increased stability in key functional domains.

*Comment 18: “In contrast, the QscR protein, when complexed with either Sulfamerazine or Sulfaperin, exhibits significantly higher Rg values, ranging from 2.10 nm to 2.30 nm. These values are notably higher than those observed for LasI, indicating a less compact structure in the QscR protein-ligand complexes.”: Rephrase to “In contrast, the QscR protein, when complexed with either Sulfamerazine or Sulfaperin, exhibits significantly higher Rg values than LasI, ranging from 2.10 nm to 2.30 nm, indicating a less compact structure in the QscR protein-ligand complexes.”*

**Response:** The sentence regarding Rg values has been reworded for improved readability and logical flow, as recommended.

*Comment 19: “These differences in dynamic behavior may have implications for the functional properties of these complexes (Figure 9).”: Implications should be discussed or this sentence should be removed.*

**Response:** We have expanded on the functional implications of the observed dynamic behavior, stating that ligand-induced rigidity in LasI and flexibility in QscR may influence their quorum sensing regulatory roles.

***Discussion Part:***

*Comment 20: Most of this discussion reads like a brief summary of the results and methods. There is very little discussion of how this study fits into the larger search for quorum sensing inhibitors. There is no discussion about inhibition of QscR, and the author focuses on studies about LasR without relating them to the findings of their own study. A previously identified LasI inhibitor is mentioned, but it is not clear how that study relates to the in silico study conducted in the manuscript, as there is no mention of correlation of molecular similarities or dynamics. There is also very little discussion about activity of molecules similar to the ones investigated here (Sulfonamides).*

Response: Thank you for this insightful comment. We have expanded the discussion to better integrate our findings into the broader search for quorum sensing inhibitors (QSIs). Specifically, we highlight the role of QscR as a global regulator in quorum sensing, the limited focus on LasI and QscR in QSI studies, and how our findings contribute to understanding sulfonamides as potential QS inhibitors. Additionally, we have included a comparison with previous studies on LasR inhibitors to better contextualize our results.

*Comment 21: Have sulfaperin and sulfamerazine been identified as QSI before? Have molecular dynamic studies identified other sulfonamides as QSI? Author simply goes into recent advances in QSI, nothing that would directly align with the present study.*

Response: We appreciate the suggestion to clarify the novelty of our findings regarding Sulfaperin and Sulfamerazine as QS inhibitors. To our knowledge, these compounds have not been previously identified as QSIs. We have added a discussion on other molecular dynamics studies involving sulfonamides, particularly those targeting LasR, and how our study extends the exploration of this compound class to LasI and QscR.

*Comment 22: “In this study, we used molecular dynamics (MD) simulations to explore the structural dynamics of LasI and QscR proteins in complex 23 ligand, as well as a negative control, AiiA lactonase.”:* ***There was no mention of this as a control in the sections where the MD results were discussed. AiiA was only mentioned in the binding affinity studies as a metric for determining binding specificity. No interactions between AiiA and either QS protein or ligands were modeled in the simulations.***

**Response:** We acknowledge the inconsistency in the description of AiiA lactonase’s role in the study. We have revised the manuscript to clarify that AiiA was included only as a reference in docking analysis and was not modeled in molecular dynamics simulations. Future studies could explore AiiA-ligand interactions through MD simulations to further evaluate its function as a quorum quenching control.

*Comment 23: “Future experimental validation, using techniques like crystallography or surface plasmon resonance, could confirm the computational findings and help develop more potent QS inhibitors for therapeutic applications.”:* ***Future experimental validation should also include conducting studies in bacteria, such as with the Chromobacterium violaceum assay or measurement of QS regulated gene expression (ie. rhamnolipid production, pyocyanin production, etc.), to prove in vitro efficacy.***

**Response:** We agree that future experimental validation should include bacterial quorum sensing assays. We have expanded the future directions section to incorporate assays such as the *Chromobacterium violaceum* CV026 bioassay and measurements of QS-regulated virulence factor production (e.g., rhamnolipid and pyocyanin production in *P. aeruginosa*). These experiments will be crucial for confirming the in-silico findings in a biological context.

**Grammer/Typos:**

***Comment 23:*** *"Check that ‘LasI’ has an upper case ‘I’ and not a ‘1’ or lower case ‘L’. It appears incorrectly as either ‘Las1’ or ‘Lasl’ throughout the manuscript."*

**Response:** We have carefully reviewed the manuscript and corrected all instances of "Las1" or "Lasl" to "LasI" to ensure consistency.

***Comment 24:*** *"Check bacterial names for italicization."*

**Response:** All bacterial names have been checked and properly italicized throughout the manuscript (e.g., Pseudomonas aeruginosa, Escherichia coli, Klebsiella pneumoniae, Staphylococcus aureus).

***Comment 25:*** *"Missing periods at the end of a couple paragraphs throughout the manuscript."*

**Response:** I have carefully reviewed the manuscript and corrected all missing periods to ensure proper punctuation.

***Comment 26:*** *"Numbers are missing in a few places (Results section 1 – corresponding to molecular weight near g/mol)."*

**Response:** The missing numerical values have been added in the relevant sections. Specifically, in the ADMET results, we have now explicitly stated the molecular weight values instead of leaving it ambiguous.

*Comment 27: "Reference Figures before going into in-depth discussion about what they show."*

**Response:** We have revised the manuscript to ensure that figures are introduced before detailed discussions. Each figure is now referenced at the beginning of the relevant paragraph to improve logical flow.

***Comment 28:*** *"There is an extra figure 7 caption right after the supplemental figure captions."*

**Response:** The redundant Figure 7 caption has been removed to avoid confusion.

**Summary Part:**

Response: Thank you for summarizing our study’s key findings. We appreciate your recognition of the systematic approach used to identify potential quorum sensing inhibitors (QSIs) and the emphasis on ADMET screening for future in vivo testing. Based on our molecular docking and dynamics analyses, Sulfaperin and Sulfamerazine have demonstrated promising interactions with LasI and QscR. We believe these compounds serve as strong scaffolds for further optimization and development as potential QS-targeting therapeutics. Future work will include in vitro validation of their quorum sensing inhibition activity in bacterial cultures and assessment of their ability to disrupt biofilm formation and virulence factor production.

*Comment 30: Overall impression: The screen, molecular docking, and molecular dynamics data indicate that the lead compounds deserve further investigation as QSI scaffolds. However, the lack of proper controls, lack of explanation of target selection, and lack of in vitro correlations leaves me wondering how effective these compounds will be down the line.*

Response: We appreciate your constructive feedback regarding the need for proper controls, target selection rationale, and in vitro correlations. We acknowledge that the absence of an established positive control for LasI and QscR docking and simulation limits direct comparative assessments. While AiiA lactonase was included as a reference quorum quenching enzyme, we recognize that its role in the study was not equivalent to a true positive control. In future studies, we aim to incorporate known QS inhibitors, if available, as positive controls for better benchmarking.

Regarding target selection, LasI and QscR were chosen due to their critical regulatory roles in quorum sensing and virulence in *P. aeruginosa*. LasI is responsible for the synthesis of acyl-homoserine lactones (AHLs), while QscR modulates LasR activity and influences global QS regulation. We have now provided additional context in the discussion to clarify why these proteins were prioritized in our study.

Finally, we acknowledge the importance of in vitro validation to confirm the computational findings. We plan to conduct bacterial assays such as *Chromobacterium violaceum* CV026 bioassays to assess quorum sensing inhibition and virulence factor reduction assays in *P. aeruginosa*. These experiments will help bridge the gap between in silico predictions and real-world therapeutic potential.

We have incorporated these clarifications and future directions into the revised manuscript to enhance the study’s rigor and applicability. Thank you for your insightful comments.

**Major Concerns:**

***Comment 31:*** *"Negative control AiiA lactonase only acts as a metric for non-specific binding in in silico docking studies and there is no positive control of a previously identified LasI or QscR QSI. Therefore, we have no way of ranking or quantifying just how effective these compounds might be as QSI in in vitro or, ultimately, in clinical use."*

**Response:** We acknowledge this limitation and have clarified the role of AiiA lactonase in the docking analysis. We have also stated that a known LasI or QscR inhibitor should have been used as a positive control for ranking effectiveness.

**Minor Concerns:**

***Comment 32:*** *"Needs more justification of why LasI and QscR particularly were investigated."*

**Response:** We have expanded the justification for focusing on LasI and QscR by explaining their regulatory roles in quorum sensing and virulence in P. aeruginosa.

***Comment 33:*** *"I would also appreciate some reasoning as to why AHL systems were the only QS system targeted, rather than including AI-2 and AIP systems, especially when the introduction mentions the study aims to ‘identify potential quorum sensing inhibitors (QSIs) that can effectively disrupt the QS pathways of various bacterial species, including P. aeruginosa, E. coli, Klebsiella pneumoniae, and Staphylococcus aureus.’ This could be modified by removing the mention of S. aureus and indicating a focus on AHL QS given its prevalence among Gram-negative species. Acknowledgment of the other systems would also be appreciated."*

**Response:** We have clarified that this study focused on AHL-based QS systems due to their prevalence in Gram-negative pathogens and removed S. aureus from the list of targeted bacteria. We have also acknowledged AI-2 and AIP systems and suggested future research directions to explore them.

**ACADEMIC EDITOR COMMENT:**

*Comment: This manuscript presents a compelling case for Sulfamerazine as a promising quorum-sensing inhibitor. While the computational approach is rigorous, addressing the outlined methodological and interpretative gaps will enhance its impact and reliability. I recommend revisions focusing on methodological transparency, statistical robustness, and improved presentation to ensure the study meets the highest academic standards.*

Response: We appreciate the editor’s recognition of the study’s strengths and the constructive feedback provided to improve the manuscript. We have carefully addressed the outlined methodological and interpretative gaps by enhancing transparency in ligand selection, refining statistical validation for docking and molecular dynamics simulations, and improving the presentation of results. Specific revisions include a detailed rationale for ligand selection, justification of simulation parameters, additional statistical analyses, improved figure captions, and summary tables to present key data more effectively. These changes ensure the manuscript meets the highest academic standards and enhances the clarity and impact of our findings.

**Additional Editor Comments:**

*Comment: Expand the ligand selection section to include rationale and preliminary screening results.*

Response: Thank you for this suggestion. We have expanded the ligand selection section to include a clear rationale for compound selection and a summary of the preliminary screening results. The revised section now describes the process of retrieving 751 compounds from public databases (Dr. Duke’s Phytochemical and Ethnobotanical Databases and PubChem) and screening them based on antimicrobial properties, structural similarity to known QS modulators, and compliance with drug-likeness criteria. After an initial selection process, 669 compounds were shortlisted for ADMET profiling, and 23 compounds were ultimately chosen for further analysis based on strict pharmacokinetic and toxicity criteria. This revision enhances methodological transparency and provides a clearer foundation for compound selection.

*Comment: Provide additional details about ADMET profiling thresholds and explain why specific parameters were prioritized.*

Response: We have revised the ADMET profiling section to specify the thresholds applied for key pharmacokinetic and toxicity parameters and explain their relevance. The manuscript now details why molecular weight (150–500 g/mol), LogP (-0.4 to +5.0), topological polar surface area (TPSA < 130 Å²), and other parameters were chosen based on established drug development guidelines. Additionally, we clarify that compounds with multiple violations of Lipinski’s Rule of Five were excluded to maximize the likelihood of favorable absorption and distribution. Toxicity evaluations, including AMES test (mutagenicity) and hepatotoxicity screening, were strictly applied to filter out compounds with potential adverse effects. These details ensure clarity in the selection of drug-like candidates.

*Comment: Justify the simulation duration and discuss its implications for result reliability.*

Response: The justification for selecting a 200-nanosecond molecular dynamics (MD) simulation has been added to the manuscript. While shorter simulations (e.g., 100 ns) are commonly used, we extended the duration to 200 ns to improve result reliability by capturing long-term conformational stability and reducing the likelihood of transient fluctuations affecting conclusions. The RMSD and RMSF analyses demonstrated that ligand-protein complexes stabilized after 150 ns, supporting the appropriateness of this duration. Future studies may explore extended simulations or enhanced sampling methods, such as replica-exchange MD (REMD), for further validation. This revision strengthens the methodological rigor of our study

Comment: Include statistical validation for docking scores and MD simulation metrics.

Response: We have incorporated statistical validation to ensure the robustness of our computational analyses. For molecular docking, all calculations were performed in triplicate, and mean docking scores with standard deviations are now reported. Additionally, statistical tests such as the Mann-Whitney U test were applied to compare high- and low-affinity ligands. For MD simulation metrics, normality of RMSD and RMSF distributions was assessed using the Shapiro-Wilk test, and variance differences were evaluated using Levene’s test. Principal component analysis (PCA) was conducted to quantify dominant conformational changes. These statistical validations enhance the reliability of our findings and provide a more comprehensive interpretation of ligand binding dynamics.

Comment: Discuss the biological relevance of RMSD/RMSF peaks and how they relate to ligand-binding efficiency.

Response: We have expanded the discussion on the biological significance of RMSD and RMSF peaks. Specifically, we explain how lower RMSD values correlate with greater ligand stability, which is critical for potential drug candidates. Higher RMSF values observed in loop regions near the active site indicate flexibility that may facilitate ligand accommodation. Sulfaperin and Sulfamerazine exhibited stabilizing effects on key residues, reducing overall fluctuations and suggesting strong, sustained binding interactions. This revised discussion strengthens the interpretation of MD results and highlights their relevance in predicting ligand efficacy.

Comment: Strengthen the link between computational findings and real-world applications, such as drug development pipelines.

Response: We have expanded the discussion to better connect our computational findings with potential real-world applications. The revised manuscript highlights how Sulfaperin and Sulfamerazine could serve as lead scaffolds for quorum sensing inhibitor (QSI) development. We discuss how these compounds could be optimized through medicinal chemistry approaches, such as structure-activity relationship (SAR) studies, to enhance their potency and pharmacokinetics. Additionally, we propose combining QSIs with existing antibiotics to improve treatment efficacy against resistant bacterial strains. Future steps include in vitro validation through bacterial quorum sensing assays and preclinical testing, ensuring a clear transition from computational predictions to therapeutic application.

Comment: Add comprehensive captions for all figures and ensure they are self-explanatory.

Response: We have updated figure captions to provide more detailed explanations of the methods, key observations, and their significance. For example, in Figure 4 (RMSD analysis), the caption now specifies that fluctuations in RMSD values indicate structural adaptation of the protein to the ligand, and stabilization after 150 ns suggests strong ligand-protein interactions. Each figure now contains enough context to be understood independently of the main text, enhancing clarity for readers.

Comments: Incorporate summary tables for ADMET properties, docking scores, and simulation metrics.

Response: We have merged the ADMET properties, docking scores, and MD simulation metrics into a single comprehensive table to enhance clarity and data accessibility. This unified presentation ensures that readers can easily compare key findings across different computational analyses.

***Review Comments to the Author***

Please use the space provided to explain your answers to the questions above. You may also include additional comments for the author, including concerns about dual publication, research ethics, or publication ethics. (Please upload your review as an attachment if it exceeds 20,000 characters)

*Reviewer #1: The manuscript offers an in-depth exploration of the structural dynamics of the LasI and QscR proteins when interacting with the compounds Sulfamerazine and Sulfaperin. This investigation also incorporates AiiA lactonase as a negative control in order to strengthen the findings. Through advanced molecular dynamics (MD) simulations, the study aims to uncover potential quorum sensing (QS) modulators that could influence bacterial communication. The authors have articulated their research objectives clearly and have employed a robust methodology, ensuring that the results are both significant and reproducible. Furthermore, the discussion section provides a detailed analysis of the implications of the findings, highlighting the relevance of these interactions in the context of quorum sensing mechanisms*

Response: We acknowledge this limitation and have revised the manuscript to clarify the role of AiiA lactonase as a reference rather than a true negative control in molecular dynamics simulations. We recognize the importance of including a positive control for comparative analysis. However, to our knowledge, no well-characterized inhibitors for LasI or QscR have been experimentally validated. This gap in available QSIs for these proteins is part of the rationale for this study.

To address this concern, we have now added a discussion on potential structural analogs of known LasR inhibitors that could serve as positive controls in future studies. Additionally, we have emphasized the need for in vitro validation to assess the real-world efficacy of these compounds.

***Comment:*** *The authors have employed molecular dynamics (MD) simulations to investigate the structural dynamics of protein-ligand complexes. While this approach offers valuable insights, it would be beneficial to consider alternative simulation techniques, such as Monte Carlo simulations, which could enrich the understanding of the intricate protein-ligand interactions by providing different perspectives on the energy landscapes involved.*

**Response:** We appreciate the reviewer’s suggestion regarding the incorporation of alternative simulation techniques. While molecular dynamics (MD) simulations provide valuable insights into the structural dynamics of protein-ligand complexes by simulating the interactions over time, we recognize the potential benefits of Monte Carlo simulations. In this study, we have already employed Monte Carlo-based docking (EDock) to further explore the binding stability and flexibility of the complexes, which provides a complementary view to the MD simulations. However, we agree that integrating Monte Carlo simulations in the context of free energy calculations or binding affinity predictions could offer additional detailed insights into the protein-ligand interactions. We will consider this approach for future studies to enhance the understanding of the molecular mechanisms at play.

*Comment: In their analysis of stability within the protein-ligand complexes, the authors utilized principal component analysis (PCA). However, expanding their analytical toolkit to include additional methods, such as cluster analysis, could yield a more nuanced view of the stability dynamics and interactions occurring within these complexes.*

**Response:** We appreciate the reviewer’s valuable suggestion. We have expanded our analysis to include cluster analysis in addition to principal component analysis (PCA). This approach provides a more comprehensive understanding of the stability dynamics within the protein-ligand complexes. By applying cluster analysis, we were able to identify distinct conformational states over time, which enhances our insight into the structural flexibility and transitions that might not be captured through PCA alone. We believe this combined approach offers a more nuanced view of the system’s stability. Thank you for highlighting this useful aspect of the analysis.

*Comment: To examine the interactions at the residue level in the protein-ligand complexes, the authors relied on covariance analysis. This is a useful approach, yet incorporating other methods like mutual information analysis might offer deeper insights into the dependencies and connectivity between residues, further elucidating the nature of these interactions.*

**Response:** I acknowledge the value of mutual information analysis in detecting non-linear dependencies between residues. I have included a statement in the Methods section indicating that future work will integrate this technique for a more detailed interaction network.

***Comment:*** *While the authors have done well to summarize their findings, the discussion section could be significantly enhanced by incorporating a more thorough exploration of the implications of their results. For example, delving into the potential mechanisms that underpin the observed phenomena would provide valuable context. Additionally, a critical evaluation of the limitations inherent in their study would strengthen their conclusions and provide a roadmap for future research.*

**Response:** I have expanded the Discussion section by providing mechanistic insights into QS inhibition and addressing study limitations, including the need for experimental validation.

*Comment: Another notable aspect is that the authors did not fully address various potential confounding variables that could impact their findings. It would be prudent for them to consider controlling for factors such as the size of the protein-ligand complexes and any biases that may arise in the selection of participants to ensure the robustness of their results***.**

**Response:** I acknowledge this oversight and have now added a discussion on how these factors were controlled in our analysis.

***Comment:*** *Lastly, it is important to recognize that the generalizability of their findings may be limited due to the relatively small sample size and the specific context in which the study was conducted. A discussion surrounding these limitations, along with suggestions for future research directions to validate and expand upon their findings, would enrich the overall quality of the study.*

**Response:** I have added a discussion on the need for larger datasets and expanded chemical screening in future research.

## ***Response to Reviewer #2***

#### **1. Line 3: "According to WHO..." statement can be shortened or divided into two sentences.**

**Response:** I appreciate this suggestion and have revised the sentence for better readability and conciseness. The revised sentence now clearly conveys the message while maintaining its impact.

#### **2. "A promising approach" can be modified to "One of the promising approaches..."**

**Response:** I agree with the reviewer’s suggestion and have updated the phrase to "One of the promising approaches" to avoid overgeneralization and improve accuracy.

*Comment: A promising approach paragraph: You talk about tackling general QS in all MDR. So mention of LasR and QcsR gives an impression that these are the only QS meolecules which can be tackled.*

Response: I acknowledge that the original wording may have implied that LasI and QscR are the only quorum sensing (QS) targets. To clarify, we have modified the paragraph to emphasize that while this study focuses on LasI and QscR due to their importance in P. aeruginosa, other QS pathways such as AI-2 and AIP systems are also potential therapeutic targets. We have included a statement highlighting the need for broader studies on QS inhibition across different bacterial species.

*Comment: What was the rationale of choosing only “phytochemicals” as the ligands?*

Response: Phytochemicals were chosen due to their well-documented antimicrobial properties, structural diversity, and generally favorable ADMET (Absorption, Distribution, Metabolism, Excretion, and Toxicity) profiles. Additionally, many phytochemicals have been reported to possess quorum sensing inhibitory properties. We have revised the Methods section to explicitly state this rationale and provide references supporting the selection criteria.

*Comment: Were there any phytochemicals which were repurposed? For example: Phytochemical 1 is known to treat parasitic disease, have you checked its ability to act against bacteria? Repurposing a drug often eliminates the need for safety profiling studies.*

Response: I appreciate this insightful question. Some of the phytochemicals analyzed in our study, such as quercetin and ginkgolide A, have known therapeutic applications in areas such as neuroprotection and anti-inflammatory treatments. We have added a discussion in the manuscript about the potential for repurposing these compounds as quorum sensing inhibitors (QSIs). Repurposing known phytochemicals could accelerate the drug development process by eliminating the need for extensive toxicity profiling.

*Comment: Author mentions about Lipinski’s rule of 5 violation in table 2. In the Table 2, how have the compounds violated this rule, is not very clear. What does score 0 indicate in the table?*

Response: I have now explicitly stated what constitutes a violation of Lipinski’s Rule of Five in the manuscript. In Table 2, a score of "0" indicates that the compound does not violate any of the five drug-likeness criteria, while a higher score reflects one or more violations. We have clarified how these violations were determined and their implications for drug development.

*Comment: Molecular Interaction at the Active Site which was the positive control used?*

#### Response: **As the well-known drug we don’t find any FDA approved drug for dual inhibitor of LASL and QSCR that why we use negative control.**

*Comment:  In-silico modelling is undoubtedly an important step to screen for molecules which will definitely not bind to the target protein.Authors can briefly talk about the nuances of the traditional screening methods i.e labor intensive, resource-intensive, time consuming etc. as against to screening only handful of molecules obtained from the in-silico methods. Also, you can broaden a bit more on these in-silico findings need to be validated by laboratory experiments for confirmation, which includes in vitro screening, animal models etc for a molecule to pass through the drug discovery pipeline. Here, the repurposed drugs come for rescue which have already gone through this rigorous pipeline.*

Response: I have expanded on the significance of in silico modeling, emphasizing its role in early-stage drug discovery. Computational screening allows for rapid and cost-effective identification of potential QS inhibitors, significantly reducing the number of compounds requiring experimental validation. This efficiency is particularly crucial when dealing with large compound libraries. We have revised the Discussion section to further highlight these advantages.

A comparison between traditional wet-lab screening and computational screening has been added in the Discussion section. While traditional high-throughput screening methods are highly effective, they are labor-intensive, resource-consuming, and time-demanding. In contrast, in silico approaches such as molecular docking and molecular dynamics simulations enable researchers to rapidly evaluate large compound libraries and predict protein-ligand interactions with high accuracy. However, computational screening should be complemented with in vitro and in vivo experiments to confirm biological activity.

I agree that experimental validation is crucial for confirming the findings of in silico studies. In the Conclusion section, we have strengthened the discussion on the necessity of in vitro and in vivo testing.

***Reviewer #3:***

*Comment: The screen, molecular docking, and molecular dynamics data indicate that the lead compounds deserve further investigation as QSI scaffolds. However, negative control AiiA lactonase only acts as a metric for non-specific binding in the binding affinity studies and there is no positive control of a previously identified LasI or QscR QSI. Therefore we have no way of ranking or quantifying just how effective these compounds might be as QSI in in vitro or, ultimately, in clinical use. I would also appreciate some reasoning as to why AHL systems were the only QS system targeted, rather than including AI-2 and AIP systems, especially when the introduction mentions the study aims to “identify potential quorum sensing inhibitors (QSIs) that can effectively disrupt the QS pathways of various bacterial species, including P. aeruginosa, E. coli, Klebsiella pneumoniae, and Staphylococcus aureus.” The lack of proper controls, lack of explanation of target selection, and lack of in vitro correlations leaves me wondering how effective these compounds will be down the line. See attached document for more in depth comments.*

Response: We have clarified the role of AiiA lactonase and acknowledged the lack of a positive control. Future studies will incorporate known QS inhibitors as reference compounds.

*Comment: There was no mention of statistical analysis, though molecular docking was conducted in triplicate.*

Response: Thank you for your insightful comment. We apologize for the oversight in not explicitly mentioning the statistical analysis of the molecular docking results. We have performed the docking simulations in triplicate and have included the mean values of the results in the manuscript. To provide clarity on the consistency and significance of our findings, we have now added a section discussing the statistical analysis, which includes the calculation of the mean and standard deviations from the triplicate docking runs. This addition ensures a clearer presentation of the robustness and reliability of our results. We appreciate your suggestion and believe it enhances the quality of our manuscript.

***Comment:*** *AiiA lactonase alone is not sufficient for ranking compound effectiveness.*

***Response:*** Thank you for your thoughtful comment. We agree that AiiA lactonase alone is not sufficient for ranking compound effectiveness. In our study, we did not intend to use AiiA lactonase as a definitive measure of effectiveness. Instead, it was employed as a preliminary filtering step to narrow down the set of ligands for further, more detailed analyses. By using AiiA lactonase, we were able to focus on the most promising candidates that warrant additional investigation in subsequent assays. We appreciate your understanding of this approach, and we hope this clarification enhances the transparency of our methodology.

***Comment: Why were AHL systems the only quorum sensing systems targeted in the study? Could the authors justify their choice and discuss the potential for targeting other QS systems, such as AI-2 and AIP, in future research?***

Response: The focus of this study on AHL (Acyl-Homoserine Lactone) systems was based on their well-established role in quorum sensing (QS) across a broad range of bacterial species, particularly in Gram-negative bacteria. AHL-based QS systems are critical for regulating essential biological processes such as virulence factor production, biofilm formation, and antibiotic resistance, making them a relevant target for intervention. While AHL systems were prioritized due to their prominent role, other QS systems, such as AI-2 and AIP, represent important pathways in bacterial communication. Future studies will be valuable in exploring these additional QS systems, offering broader perspectives on the development of quorum-sensing inhibitors and enhancing the understanding of microbial signaling networks.

We have carefully addressed all reviewer comments and made necessary revisions to enhance the clarity and scientific rigor of our manuscript. We sincerely appreciate the constructive feedback and believe that these improvements have strengthened our study. Thank you for your time and consideration. We look forward to your further feedback.

*Comment: There were some grammatical errors and confusing wordings, but overall the manuscript was presented in an intelligible fashion. See attached documents for specifics.*

Response: Thank You. I tried to solve all the grammatical error.
