# Supplementary material for: In silico analysis of quorum sensing modulators: Insights into molecular docking and dynamics and potential therapeutic applications
Source: PLoS One. 2025 Jun 9;20(6):e0325830. doi: 10.1371/journal.pone.0325830 (PMC12148138; doi:10.1371/journal.pone.0325830)
Supplement: Attachment — Submitted filename: Response to Reviewers.docx [file pone.0325830.s010.docx]

**Response to Reviewers
Manuscript ID: PONE-D-25-00287R1
Title: *In Silico Analysis of Quorum Sensing Modulators: Insights into Molecular Docking and Dynamics and Potential Therapeutic Applications*
Journal: *PLOS ONE***

**We sincerely thank the academic editor and reviewers for their time, thoughtful comments, and constructive feedback. We appreciate the recognition of our manuscript’s relevance in addressing antimicrobial resistance through alternative therapeutic strategies and have revised the manuscript accordingly to address all concerns raised. Below, we provide a detailed, point-by-point response to each comment. Reviewer comments are shown in bold, followed by our responses in *regular font*.**

**Reviewer #1: *The manuscript presents a comprehensive in silico analysis of quorum sensing (QS) modulators, focusing on the structural dynamics of LasI and QscR proteins in Pseudomonas aeruginosa. The study employs molecular docking and molecular dynamics (MD) simulations to evaluate the binding affinity and stability of potential QS inhibitors (QSIs), with a particular focus on Sulfamerazine and Sulfaperin. The manuscript is well-structured and addresses an important area of research, given the rising concern of antimicrobial resistance (AMR) and the need for alternative therapeutic strategies. However, there are several areas where the manuscript could be improved to enhance its scientific rigor, clarity, and impact.***

**We sincerely thank the reviewer for their detailed and constructive feedback. We greatly appreciate the acknowledgment of the manuscript's structure and its relevance in addressing antimicrobial resistance (AMR) through alternative therapeutic strategies. In response to the suggestions aimed at enhancing the scientific rigor, clarity, and impact of our study, we have thoroughly revised the manuscript to address the concerns raised.**

**Reviewer’s Comment 1:**
***While the in-silico approach is valuable for initial screening, the absence of experimental validation (e.g., in vitro assays) limits the study's impact. The authors should acknowledge this limitation more explicitly and discuss plans for future experimental validation, such as bacterial quorum sensing assays (e.g., Chromobacterium violaceum CV026 bioassay) or virulence factor inhibition assays in P. aeruginosa.***
**Response:**
We agree with the reviewer. The limitation regarding the absence of experimental validation has now been explicitly acknowledged in the revised manuscript (Discussion section, after the sentence: “…providing a foundation for future drug design”). In addition, a paragraph has been added outlining future plans to conduct bacterial quorum sensing assays, including the *Chromobacterium violaceum* CV026 bioassay and virulence factor inhibition assays in *Pseudomonas aeruginosa*, to experimentally validate the in-silico predictions.

**Reviewer’s Comment 2:
*The manuscript does not provide a strong rationale for selecting Sulfamerazine and Sulfaperin as the primary ligands. While the ADMET profiling is thorough, more context is needed regarding why these specific compounds were chosen over others. For example, are these compounds known to have antimicrobial or QS inhibitory properties in previous studies?*
Response:**
Thank you for pointing this out. We have expanded the “Selection of Ligand” subsection in the Methods and Materials section to clarify that Sulfamerazine and Sulfaperin were selected based on their favorable ADMET profiles, structural similarity to known antimicrobial sulfonamides, and strong binding scores from molecular docking. This clarification has been inserted after the line referencing Table 2.

**Reviewer’s Comment 3:**
***The discussion section could be expanded to include a more critical evaluation of the study's findings in the context of existing literature. For instance, how do the binding affinities and stability of Sulfamerazine and Sulfaperin compare to other known QSIs? Additionally, the authors should discuss the potential challenges in translating these findings into clinical applications, such as bioavailability, toxicity, and resistance development.***
**Response:**
We appreciate this valuable suggestion. The Discussion section has been enhanced to include a comparison of the binding affinities of Sulfamerazine and Sulfaperin with known QSIs such as TZD-C8, which has demonstrated both in vitro activity and comparable or stronger docking scores. Furthermore, we have incorporated a discussion on potential translational challenges, including bioavailability, toxicity, and the possibility of resistance development, which may affect clinical applicability.

**Reviewer’s Comment 4:**
***The use of AiiA lactonase as a negative control is somewhat confusing. While the authors explain that AiiA was used for reference docking, it is not clear how this serves as a negative control in the context of MD simulations. A more appropriate negative control would have been a known non-inhibitor or a scrambled peptide. This should be clarified in the manuscript.***
**Response:**
We agree that the explanation provided earlier may have been unclear. The Discussion section has now been revised to clarify that AiiA was used solely as a reference in docking due to its known quorum-quenching ability and not as a negative control in MD simulations. We acknowledge that a known non-inhibitor or scrambled peptide would serve as a more appropriate negative control and have noted this as a limitation to be addressed in future work.

**Reviewer’s Comment 5:**
***The manuscript mentions that docking simulations were performed in triplicate, but there is no detailed statistical analysis to support the robustness of the results. Including statistical tests (e.g., Mann-Whitney U test, Shapiro-Wilk test) would strengthen the validity of the findings.*
Response:**
Thank you for this important comment. We have now included statistical validation methods in the Molecular Dynamics Simulation analysis section. Specifically, the Mann-Whitney U test was applied to compare docking scores, thereby enhancing the robustness and reliability of the reported data.

**Reviewer’s Comment 6:**
***The study focuses exclusively on AHL-based QS systems in Gram-negative bacteria. While this is justified, the authors should briefly discuss the potential applicability of their findings to other QS systems (e.g., AI-2 or AIP systems) and Gram-positive bacteria. This would broaden the scope and relevance of the study.***
**Response:**
We appreciate the reviewer’s insightful suggestion. We have addressed this in the Conclusion section by including a brief discussion on the broader applicability of quorum sensing inhibitors, including their relevance to AI-2 and AIP-based systems in both Gram-negative and Gram-positive pathogens.

**Reviewer #3**

**Reviewer’s Comment 1:**
***Minor concerns about justification of investigating LasI and QscR, focusing on AHL systems only, and acknowledgment of other QS systems was adequate.*
Response:**
Thank you for acknowledging our rationale. We appreciate that the focus on LasI and QscR within AHL-based quorum sensing systems was considered adequate. To further strengthen the manuscript, we have included a short discussion on the relevance of our findings to other quorum sensing systems such as AI-2 and AIP, particularly in Gram-positive bacteria like *Staphylococcus aureus*.

**Reviewer’s Comment 2:**
***The claim "Quorum sensing inhibition has been explored as an alternative to conventional antibiotics, with numerous QSIs identified from natural and synthetic sources" needs citations.***
**Response:**
Thank you for your observation. We have now added appropriate references in the Introduction to support this claim, including recent studies reporting synthetic compounds (e.g., TZD-C8) and natural products as quorum sensing inhibitors.

**Reviewer’s Comment 3:**
***Table 1 is never referenced in the text.***
**Response:**
This oversight has been corrected. Table 1 is now cited in the ADMET Profiling section following the description of screening criteria for pharmacokinetics and toxicity.

**Reviewer’s Comment 4 & 5:
*Major concerns about the study design and lack of proper controls to support the study findings were not adequately addressed. The author mentions AiiA is used only to show low binding affinity and is not a control for MD studies. While they say no positive controls are available, TZD-C8 is later mentioned with QS inhibition properties and binding affinities. The author could include these values as metrics for benchmarking and compare with other studies. The reviewer argues that these controls are required and the study currently lacks them.*
Response:**
We appreciate the reviewer’s critical observations regarding the absence of appropriate controls. In response, the revised manuscript now includes two well-characterized quorum sensing inhibitors as positive controls in our docking studies:

1. **TZD-C8 (Z-5-octylidene-thiazolidine-2,4-dione)** is included as a reference LasI inhibitor based on validated in vitro and in silico quorum sensing inhibition (referenced in Refs. 21–23).
2. **N-dodecanoyl-L-homoserine lactone**, a natural signaling molecule with high affinity for QscR, is used as a positive ligand control (Refs. 21–23).

These compounds have been incorporated into the docking experiments (Table 3), and their binding affinities (–7.4 kcal/mol and –7.5 kcal/mol, respectively) are reported. Their inclusion serves as a benchmark for evaluating our screened ligands and validates our docking protocol. The Discussion section has been updated accordingly to reflect their relevance and to contextualize our novel ligand findings. We trust this revision addresses the concern regarding controls and improves the study’s scientific rigor.
